# Correlation between *Stenotrophomonas maltophilia* incidence and systemic antibiotic use: A 10-year retrospective, observational study in Hungary

**DOI:** 10.1556/1886.2024.00022

**Published:** 2024-03-05

**Authors:** Márió Gajdács, Mária Matuz, Benkő Ria, Zoltán Pető, Edit Hajdú

**Affiliations:** 1 Department of Oral Biology and Experimental Dental Research, Faculty of Dentistry, University of Szeged, 6720 Szeged, Tisza Lajos krt. 64-66., Hungary; 2 Central Pharmacy Department, Albert Szent-Györgyi Medical Center, University of Szeged, 6725 Szeged, Semmelweis utca 6., Hungary; 3 Institute of Clinical Pharmacy, Faculty of Pharmacy, University of Szeged, 6725 Szeged, Szikra utca 8., Hungary; 4 Department of Emergency Medicine, Albert Szent-Györgyi Medical Center, University of Szeged, 6725 Szeged, Semmelweis utca 6., Hungary; 5 Department of Internal Medicine Infectiology Unit, Albert Szent-Györgyi Clinical Centre, University of Szeged, 6725 Szeged, Állomás Street 1–3, Hungary

**Keywords:** Stenotrophomonas maltophilia, antimicrobial resistance, antibiotic consumption, carbapenems, selection pressure, antimicrobial stewardship

## Abstract

Extensive use of carbapenems may lead to selection pressure for *Stenotrophomonas maltophilia* (SM) in hospital environments. The aim of our study was to assess the possible association between systemic antibiotic use and the incidence of SM. A retrospective, observational study was carried out in a tertiary-care hospital in Hungary, between January 1st 2010 and December 31st 2019. Incidence-density for SM and SM resistant to trimethoprim-sulfamethoxazole (SXT) was standardized for 1000 patient-days, while systemic antibiotic use was expressed as defined daily doses (DDDs) per 100 patient-days. Mean incidence density for SM infections was 0.42/1000 patient-days; 11.08% were were resistant to SXT, the mean incidence density for SXT-resistant SM was 0.047/1000 patient-days. Consumption rate for colistin, glycopeptides and carbapenems increased by 258.82, 278.94 and 372.72% from 2010 to 2019, respectively. Strong and significant positive correlations were observed with the consumption of carbapenems (r: 0.8759; *P* < 0.001 and r: 0.8968; *P* < 0.001), SXT (r: 0.7552; *P* = 0.011 and r: 0.7004; *P* = 0.024), and glycopeptides (r: 0.7542; *P* = 0.012 and r: 0.8138; *P* < 0.001) with SM and SXT-resistant SM incidence-density/1000 patient-days, respectively. Implementation of institutional carbapenem-sparing strategies are critical in preserving these life-saving drugs, and may affect the microbial spectrum of infections in clinical settings.

## Introduction

Non-fermenting Gram-negative bacteria are a heterogenous group of Gram-negative rods in the Pseudomonadota phylum [1]; they are characterized by the lack of enzymes needed to ferment sugars, and their omnipresence in aquatic habitats, soil, and in healthcare-associated environments [2]. Following *Pseudomonas aeruginosa, Acinetobacter baumannii* complex and *Burkholderia cepacia* complex, *Stenotrophomonas maltophilia* (SM) is the fourth most commonly isolated species from clinical specimens among non-fermenters [3]. SM has been described as a low-grade pathogen with limited invasiveness: natural host defences must be circumvented for this microorganism to cause an infection, thus, these infections are often described in immunocompromised patients [4]. On the other hand, they are potent producers of biofilm, outer membrane vesicles and siderophores, they have a charged cell surface, and they possess tissue-degrading enzymes, a variety of efflux pumps, nonfimbrial adhesins and protein secretion systems (i.e. a type II [Xps] and a type IV [VirB/D4] secretion system) [2, 3, 5, 6]. These virulence factors allow for the survival and persistence of SM in harsh environmental conditions in hospital settings, and shield the microorganism from the immune system and antimicrobials *in vivo* [2, 3, 7].

Before the 1980s, the isolation of SM from true infections was rare [8]; however, since then, SM is increasingly being recognized as an important etiological agent in healthcare-associated infections (HAIs), with an incidence of 0.70–3.77 cases/1000 discharges, according to literature sources [9, 10]. This may be explained by the higher number of patients at risk, more advanced diagnostic methods in microbiology, in additon to developments in invasive surgical interventions and the therapy of malignant disorders [11]. In addition, since the 2000s, reports of SM as a pathogen in community-acquired infections have also emerged [12]. SM is an important etiological agent in tracheobronchitis/pneumonia and bacteremia/sepsis; both manifestations were associated with a high overall mortality rate (25–75% and 20–60%, respectively) [9, 13, 14]; a fulminant manifestation of fatal hemorrhagic pneumonia has also been described [15]. Nevertheless, the clinical relevance of SM was also noted in skin and soft tissue infections, invasive bone and joint infections, ocular infections, meningitis, endocarditis and urinary tract infections [16]. Risk factors for invasive SM infections include severe immunosuppression (either due to a human immunodeficiency virus [HIV] infection or cancer treatments), neutropenia, admission to the intensive care unit (ICU), mechanical ventilation, recent surgery or traumatic event, parenteral nutrition and history of broad-spectrum antimicrobial therapy or colonization with SM [17]. SM is also commonly associated with outbreaks at ICUs, underscoring its importance as a critical concern for infection prevention and control [18].

The treatment of SM infections is a considerable challenge for clinicians, as these microorganisms possess intrinsic resistance mechanisms against many of the currently available antimicrobials in everyday use: they are non-susceptible to most of the β-lactam antibiotics, aminoglycosides and fosfomycin [19, 20]. Most notably, SM are resistant to carbapenems – which are often the last safe and effective treatment option in against Gram-negative infections – due to the possession of chromosomally-encoded carbapenemases (i.e. a class A serine β-lactamase [*bla*_L2_] and a class B metallo β-lactamase [*bla*_L1_]) [21]. At present, the drug of choice to treat these infections is sulfamethoxazole/trimetoprim (co-trimoxazole; SXT) at 15 mg/kg/day, divided into 3–4 doses [22]. SM infections are often treated empirically (especially in the severely immunocompromised), during which, the first-line drug is combined with fluoroquinolones (ciprofloxacin, levofloxacin and moxifloxacin), minocycline, colistin, ceftazidime, cefepime, ticarcillin/clavulanate, rifampin or chloramphenicol, although established breakpoints for antimicrobial susceptibility testing are only available for SXT [23]. Nevertheless, due to the possession of class 1 integrons (*sul1*, *sul2* and *dfrA* genes) and/or the overexpression of resistance-nodulation-division (RND)-type efflux pumps, resistance to SXT in SM (or a history of hypersensitivity in the patient) is a serious challenge for treating physicians [24]. Although a systematic review and meta-analysis by Ko et al. – which included retrospective cohort and case-control studies – demonstrated that fluoroquinolone therapy was non-inferior to SXT in SM infections (in fact, in some instances, fluoroquinolones were associated with survival benefit over SXT), further studies are needed to establish reliable evidence in this field [25]. SXT-resistance rates show considerable variation worldwide: based on a recent systematic review and meta-analysis by Dadashi et al., the highest pooled resistance rates were reported in Asia (19.29%), followed by Europe (10.52%), and the Americas (7.01%) [26]; nevertheless, individual prevalence studies from various geographical regions (e.g., Turkey: 10–15%, Spain >25%, China: 30–48%) and patient-groups (e.g., in cystic fibrosis patients: as high as 80%) may report considerably higher rates of resistance [27, 28]. HAIs caused by SXT-resistant SM are notifiable diseases under the framework of the National Nosocomial Infections Surveillance Network in Hungary (NNSzR); according to recent reports, the incidence-density of SXT-resistant SM nationally were 0.0014/1000 patient-days and 0.002/1000 patient-days in 2019 and 2020, respectively [29].

Due to the emergence and rapid global spread of extended-spectrum β-lactamases (ESBLs) in members of the Enterobacterales order, and the high rate of associated infections, there has been a considerable shift towards the more frequent use of drugs that were previously considered as „last-resort” antibiotics [19, 30]. One such group are the carbapenems (i.e. imipenem, meropenem, ertapenem and doripenem), which are often the last safe and effective therapeutic choices in these infections. The correlation between antibiotic use (especially if injudicious) and the prevalence of antimicrobial resistance has been described by numerous studies [31, 32]. In addition, shifts in systemic antibiotic consumption may have considerable effects on the epidemiology of infectious agents, both locally and globally [33]. It has been suggested that the extensive use of carbapenems (due to the omnipresence of ESBLs) may lead to selection pressure for SM – as these bacteria are intrinsically resistant – in hospital environments [34, 35]. Furthermore, previous treatment with carbapenems is a notable risk factor for colonization with SM [19, 36]. These studies may be relevant from the standpoint of aiding antimicrobial stewardship, carbapenem-sparing strategies, and infection control interventions [37]; however, no similar studies were carried out in Hungary. Thus, the aim of the present study was to assess the possible association between systemic antibiotic use (with special focus on carbapenems) and the incidence of SM over a 10-year period, preceding the coronavirus disease 2019 (COVID-19) pandemic period, in the context of a Hungarian health centre.

## Materials and methods

### Study design and location

The present retrospective, observational study was carried out at the Albert Szent-Györgyi Health Centre (HC), which is a 1820-bed primary- and tertiary-care teaching hospital, situated in Szeged, in the Southern Great Plain of Hungary. The bed capacity of the hospital at the time of the study included 1465 acute and 355 chronic beds, serving over 400,000 patients in the region annually, based on the National Health Insurance Fund of Hungary (NEAK) [38].

### Data collection, studied variables

Data collection has been carried out for the 10-year period between the 1st of January 2010 and 31st of December 2019, corresponding to the following variables at the HC: a) isolation frequency and sample type of clinically-relevant SM isolates, b) frequency of resistance to first-line treatment (SXT) in clinically-relevant SM isolates, c) patient turnover rates, d) systemic antibiotic use at the HC-level. Case definitions for clinically-relevant SM isolates were used as described previously [39]. Data on patient turnover rates at the HC and for individual departments was collected from the public database of the NEAK [38].

### Isolation, identification and antimicrobial susceptibility testing of SM isolates

Microbiological sampling and laboratory processing of the samples were carried out according to the current clinical recommendations relevant to each individual sample type. The following sample types were considered during our analysis: respiratory samples (including bronchoalveolar lavage, mini bronchoalveolar lavage [miniBAL] and tracheal aspirates), invasive samples (including blood culture samples, drains, biopsies and surgical samples), and urine samples (including midstream, catheter-specimen urine and suprapubic bladder aspirates). Identification of SM was based on VITEK 2 ID cards (bioMérieux, Marcy-l’Étoile, France) and matrix-assisted laser desorption/ionization time-of-flight mass spectrometry (MALDI-TOF MS; Bruker Daltonics, Bremen, Germany). Sample preparation protocols and the technical details of the MALDI-TOF measurements were described elsewhere [40]. The MALDI Biotyper RTC 3.1 software (Bruker Daltonics, Bremen, Germany) and the MALDI Biotyper Library 3.1 were used for spectra analysis. Antimicrobial susceptibility testing for SXT was performed using the disk diffusion method (Oxoid, Basingstoke, UK) on Mueller-Hinton agar plates (bioMérieux, Marcy-l’Étoile, France); the interpretative criteria was based on the European Committee on Antimicrobial Susceptibility Testing (EUCAST) breakpoints relevant at the time of processing [41]. Microbiological data was collected from the MedBakter laboratory information system. Isolates considered as colonizers or noted as contaminants were excluded from data analysis. Incidence-density (i.e. number of observed events divided by population-time at risk) for the isolation of SM was expressed per 1000 patient-days, in addition, frequency (*n*, %) and incidence-density of SXT-resistant isolates (per 1000 patient-days) were also recorded [42].

### Systemic antibiotic use

Antibiotic use data for the HC was collected from the Central Pharmacy Department, University of Szeged. Based on previous studies [34, 35], data for the following antibacterial groups was retrieved: antibacterials for systemic use (overall) (J01), penicillins with extended spectrum (J01CA), penicillin combinations including β-lactamase inhibitors (J01CR), second-generation cephalosporins (J01DC), third-generation cephalosporins (J01DD; including ceftazidime [J01DD02] alone), carbapenems (J01DH), SXT (J01EE01), aminoglycosides (J01G), glycopeptide antibacterials (J01XA), relevant fluoroquinolones (i.e. the summized consumption of ciprofloxacin (J01MA02), levofloxacin (J01MA12) and moxifloxacin (J01MA14)), colistin (J01XB01), and overall consumption of all other antibacterial groups (i.e. subtracting all above listed antibiotic subgroups from J01). Antibiotic use was calculated based on the World Health Organization (WHO) Collaborating Centre for Drug Statistics Methodology, and expressed as defined daily doses (DDDs) per 100 patient-days [43].

### Statistical analysis

Database creation and descriptive statistical analysis (quantities (n) and percentages (%) for categorical variables, while means with standard deviations [SD] and ranges for continuous variables) was carried out using Microsoft Excel 2013 (Redmond, WA, USA, Microsoft Corp.). Statistical analyses were performed with SPSS software version 22 (IBM SPSS Statistics for Windows 22.0, Armonk, NY, USA, IBM Corp.). Normality of variables was tested using the Shapiro-Wilk tests (data not shown). The relationship between studied variables was tested using Pearson-correlation; correlation coefficients (r) were evaluated as follows: |r|<0.3 weak or no correlation, 0.3≤|r|<0.5 moderate correlation, 0.5≤|r|<0.85 strong correlation, |r|>0.85 very strong correlation. During trend analysis, the slope (b) was determined, and the coefficient of determination (*R*^2^) was shown to assess goodness of fit. Statistical significance for all analyses was set at *P* < 0.05.

### Ethical considerations

The study was conducted in accordance with the Declaration of Helsinki and national and institutional ethical standards. As the study used aggregated data only – individual patient data was not collected and data anonymity was maintained – it was not subject to ethics review. Informed consent was not relevant as data anonymity was maintained.

## Results

### Epidemiology of SM and SXT-resistant SM

**Fig. 1.**
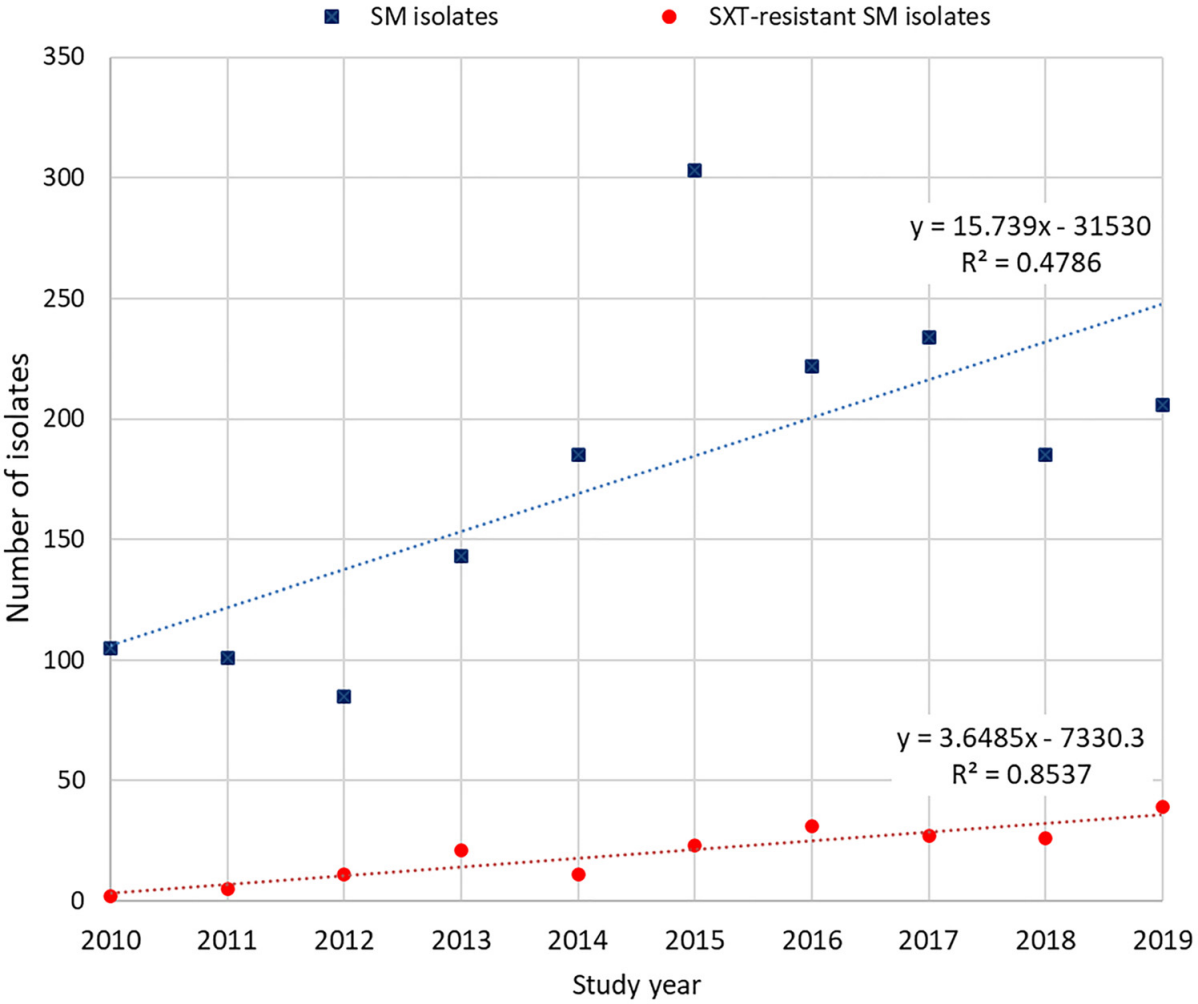
Epidemiology of SM and SXT-resistant SM isolates at the Albert Szent-Györgyi Health Centre during the study period, 2010–2019 SM: *Stenotrophomonas maltophilia*; SXT: sulfamethoxazole/trimetoprim; y: regression equation; *R*^2^: coefficient of determination.

Our data analysis covered a total of *n* = 1769 (mean ± SD: 176.90 ± 68.88/year; range: 85–303) clinically-relevant SM isolates during the 10-year study period; the mean incidence density for SM was 0.42/1000 patient-days (range: 0.19–0.73) (Fig. 1). The distribution of SM according to sample types was the following: 56.53% (*n* = 1000) from respiratory samples, 35.21% (*n* = 623) from invasive samples, *n* = 72 (4.07%) from urine samples, and *n* = 74 from other sources (4.19%).

Out of the above SM isolates, *n* = 196 (mean ± SD: 19.60 ± 11.96/year; range: 2–39) SM isolates were resistant to SXT, which corresponds to 11.08% (range: 1.90–18.93%) of isolates overall (Fig. 1); the mean incidence density for SXT-resistant SM was 0.047/1000 patient-days (range: 0.004–0.069). Both the incidence of SM and SXT-resistant SM showed a significantly increasing trend throughout the study period (*b* = 15.739, *P* = 0.027, *R*^2^ = 0.4786 and *b* = 3.649, *P* < 0.001, *R*^2^ = 0.8537, respectively), with peaks in 2015 and 2019, respectively; the secular trends in the epidemiology of SM and SXT-resistant SM throughout the study are presented in Fig. 1.

### Antibiotic use, correlation analysis

From 2010 to 2019, systemic antibiotic use (J01) has increased by 34.39%; with the exception of the relevant fluoroquinolones for the treatment of SM ([J01MA02 + J01MA12 + J01MA14] consumption decreased by 12.57%) and aminoglycosides ([J01G] consumption decreased by 13.02%), consumption of all other antibiotic groups increased during the 10-year period. The relative hike in antibiotic use was highest for third-generation cephalosporins ([J01DD] 100.09%), colistin ([J01XB01] 258.82%), glycopeptide antibacterials ([J01XA] 278.94%) and carbapenems ([J01DH] 372.72%), respectively.

The relationship between systemic antibiotic use and the epidemiology of SM and SXT-resistant SM at the HC during the study period – expressed as correlation coefficients (r) – is presented in Table 1. Overall consumption of antibacterials for systemic use (J01) showed strong and significant positive correlations with the incidence-density of SM, and SXT-resistant SM, respectively (Table 1). When considering the incidence-density for SM/1000 patient-days, strong and significant positive correlations were observed with the consumption of carbapenems, SXT, glycopeptides and the overall consumption of all other antimicrobials (Table 1). Beta-lactam antibiotics – other than carbapenems – showed moderate, non-significant correlation with the epidemiology of SM (Table 1). With the exception of penicillins with extended spectrum and aminoglycosides, correlation was positive in respect to all other antibiotic groups studied (Table 1). When analyzed separately, ceftazidime use or fluoroquinolones (ciprofloxacin, levofloxacin, moxifloxacin) did not show significant correlations with SM incidence-density (Table 1).

**Table 1.** Correlations between systemic antibiotic use and incidence-density for SM, and SXT-resistant SM at the Albert Szent-Györgyi Health Centre during the study period, 2010–2019

	Incidence-density for SM/1000 patient-days	Incidence-density for SXT-resistant SM/1000 patient-days
J01: Antibacterials for systemic use	**r: 0.7244**	**r: 0.8372**
	*P* = 0.018	*P* = 0.005
J01CA: Penicillins with extended spectrum	r: −0.5617	r: −0.1767
	*P* = 0.087	*P* = 0.625
J01CR: Penicillin combinations including β-lactamase inhibitors	r: 0.5334	**r: 0.7411**
	*P* = 0.158	*P* = 0.014
J01DC: Second-generation cephalosporins	r: 0.4283	**r: 0.7482**
	*P* = 0.304	*P* = 0.007
J01DD: Third-generation cephalosporins	r: 0.5717	**r: 0.7786**
	*P* = 0.128	*P* = 0.013
J01DH: Carbapenems	**r: 0.8759**	**r: 0.8968**
	*P* < 0.001	*P* < 0.001
J01EE01: Sulfamethoxazole and trimethoprim	**r: 0.7552**	**r: 0.7004**
	*P* = 0.011	*P* = 0.024
J01G: Aminoglycosides	r: −0.2408	r: −0.2927
	*P* = 0.508	*P* = 0.401
J01XA: Glycopeptide antibacterials	**r: 0.7542**	**r: 0.8138**
	*P* = 0.012	*P* < 0.001
Relevant fluoroquinolones (J01MA02: ciprofloxacin + J01MA12: levofloxacin + J01MA14: moxifloxacin)	r: 0.4319	r: 0.037
	*P* = 0.185	*P* = 0.911
J01XB01: Colistin	r: 0.4329	r: 0.4002
	*P* = 0.144	*P* = 0.181
Overall consumption of all other antibiotic groups	**r: 0.7989**	**r: 0.7644**
	*P* < 0.001	*P* = 0.010

SM: *Stenotrophomonas maltophilia*; SXT: sulfamethoxazole/trimetoprim; Pearson correlation coefficients (r) with *P*-values <0.05 are presented in **boldface**.

When considering the incidence-density for SXT-resistant SM/1000 patient-days, strong and significant positive correlations were observed with the consumption of carbapenems, glycopeptides, third-generation cephalosporins, second-generation cephalosporins, penicillin-combinations, including beta-lactamase inhibitors, SXT and the overall consumption of all other antimicrobials (Table 1). With the exception of penicillins with extended spectrum and aminoglycosides, correlation was positive in respect to all other antibiotic groups studied (Table 1). When analyzed separately, ceftazidime use showed strong and significant correlation, while fluoroquinolones did not show significant correlations with SXT-resistant SM incidence-density, respectively (Table 1).

## Discussion

The aim of our longitudinal study was to assess the possible association between systemic antibiotic use (with special focus on carbapenems) and the epidemiology of SM infections (including SXT-resistance) in a tertiary-care hospital in Hungary, over a 10-year period, preceding the COVID-19 pandemic. To the best of our knowledge, this is the first such study, focusing on SM in Hungary. Infections caused by SM were previously infrequently described in the literature, however, it is reasonable to expect that the relevance of SM – both in HAIs and in community settings – will continue to increase, due to population aging, higher number of individuals affected by malignancy, and more invasive medical procedures being performed with the advances in medical technologies [44, 45]. On the other hand, as a result of the COVID-19 pandemic, sharp increases in the prevalence of all MDR pathogens were noted worldwide, which will undoubtedly affect the rate of ESBL-producers as well, leading to additional pressures to use carbapenems in the clinical practice [46, 47]. As data from observational studies is scarce in Hungary (which limits reliable comparisons), our results provide novel insights into the epidemiology of SM infections.

Overall, an increasing trend was observed for the incidence-density of SM and SM resistant to the first-line drug (with peaks in 2015 and 2019, respectively). Overall systemic antibiotic consumption (J01G), and the consumption of carbapenems (J01DH), SXT (J01EE01) and glycopeptide antibiotics (J01XA) showed strong and significant correlations with both SM and SXT-resistant SM, respectively. As broad-spectrum antimicrobial therapy in the patient's history is a known risk factor for SM infections, it is reasonable to assume a possible correlation between carbapenem use with the increasing frequency of SM and SXT-resistant SM. However, the relationship between glycopeptide antibiotic use and SM epidemiology is a curious finding, as these drugs are only effective in the treatment of Gram-positive bacteria; on the other hand, their increasing use may serve as a proxy measure for the epidemiology of HAIs caused by Gram-positives (especially for methicillin-resistant *Staphylococcus aureus* and *Clostridioides difficile*), individuals in particularly severe condition or in advanced disease stages, or antibiotic combination therapy in our healthcare setting [48, 49].

In the prospective study of Sanyal and Mokaddas [34], the correlation between SM incidence, carbapenem-consumption and patient turnover rates were assessed over a 5-year period in Kuwait: similarly to our results, they found strong and significant (r: 0.97, *P* = 0.004) correlation between SM and carbapenem use, while a similar relationship was not noted for patient turnover rates. On the other hand, those isolates were pan-susceptible to SXT and ciprofloxacin, while a considerable number were declared resistant to piperacillin and ceftazidime [34]. As a part of the SARI (Surveillance of Antimicrobial Use and Antimicrobial Resistance in German Intensive Care Units) project, Meyer et al. assessed the factors affecting SM incidence, including hospital structural parameters, patient parameters and antibiotic use in 39 participating ICUs: in their study, SM represented 1.7% of all ICU isolates, with a median incidence density of 1.4 per 1000 patient-days. Length of hospital stay, urinary-catheter-days, central venous catheter (CVC)-days and ventilator-days per 100 patient-days all showed strong and significant correlations with SM incidence-density, in addition, in hospitals where there were >12 ICU beds, these infections were also significantly more common. Regarding systemic antibiotic use, their study demonstrated strong and significant correlation between consumption of carbapenems, glycopeptides and total antibiotic use with SM incidence-density; in contrast to our findings, however, they also showed a significant relationship for ceftazidime and fluoroquinolone use, while this was not the case for SXT. Overall, in their multivariate regression model, the study concluded that carbapenem use and ICU bed numbers were independent risk factors [35]. On the other hand, the relevance of carbapenems in exerting the selection pressure for SM was not verified by Carmeli and Samore [50], where SM acquisition rates in patients treated with ceftazidime and imipenem did not differ considerably, but was significantly higher in patients receiving both drugs [50]. In addition, the study of Ueda et al. [50] – where both non-fermenting Gram-negatives and Enterobacterales were included in the analysis – showed that not overall antibiotic use, but rather the low heterogeneity in antibiotic use (expressed in the antibiotic heterogeneity index [AHI]) is the driver for selective pressure and increasing rates of resistance [51].

Our study has shown SXT-resistance rates of ∼11%, which is similar to previous findings from Central and Eastern European Countries, but higher than those from Western Europe (2–10%) [26]. However, the continuous increase in the number of non-susceptible isolates is cause for concern. SXT-resistance may develop through a variety of molecular mechanisms, in which case, other therapeutic modalities need to be considered [52]. In the systematic review and meta-analysis of Prawang et al., favourable outcomes were noted for combination therapy, however, during subgroup analysis, combination therapy was associated with higher mortality risk in specific patient groups [53]. Nevertheless, SM may harbor other acquired resistance determinants against the other antibiotics left for consideration, thus, easily becoming extensively drug resistant (XDR) – according to EUCAST guidelines – within one or two steps [13, 14, 19, 26, 54]. However, no recommendation or consensus exists to guide susceptibility testing and interpretation for other possible antimicrobials, other than SXT. For this reason, there is a scarcity of data to assess the relevance of various other drugs and combination therapies. In such studies, species-specific breakpoints for other microorganisms (e.g., *A. baumannii*, *P. aeruginosa*) or non-species specific (NSS) breakpoints are used for the interpretation of results [55]. In a three-year study of Juhász et al., infective and colonizing SM isolates were subjected to susceptibility testing and clinical data collection: 99%/98%, 24%/12%, 75%/84%, 87%/90%, 12%/35% and 9%/23% of infective and colonizing isolates were susceptible to SXT, ciprofloxacin, levofloxacin, moxifloxacin, tigecycline and colistin, respectively. All-cause mortality rate was 45%, with CVCs and vasopressor therapy identified as independent risk factors for mortality [39]. While in a previous laboratory-based study at our HC, involving infective and colonizing SM isolates, phenotypic susceptibility was highest for levofloxacin and colistin (92.2%), followed by tigecycline (90.5%), SXT (87.4%) and amikacin (27.5%); 24.1% were susceptible to all tested antimicrobials, while 2.2% of isolates were classified as XDR [56]. On the other hand, a recent review has described *S. maltophilia* as intrinsically resistant to all aminoglycosides, including amikacin, kanamycin, neomycin and tobramycin [19]. Furthermore, as described by the meta-analysis of Dadashi et al., pooled resistance rates worldwide are highest for levofloxacin (14.4%), SXT (9.2%) and minocycline (1.4%), partly due to the fact that these drugs are the most commonly reported by respective studies [26].

Carbapenem-resistant (CR) Gram-negative bacilli are a daunting prospect for clinicians and for public health worldwide, due to the scarce treatment options left for their management [57]. The WHO has declared CR *A. baumannii*, *P. aeruginosa* and Enterobacterales as „Priority 1: Critical” for the development of novel antimicrobials [58]. After the increasing prevalence of ESBLs, carbapenems have become the treatment-of-choice for these infections, which unfortunately has led to the inevitable occurrence of carbapenem-resistant isolates, out of which, the acquired, plasmid-borne carbapenem-resistance is the most concerning, as these resistance genes may readily spread among different species and geographical regions worldwide [59]. Furthermore – as demonstrated by our results – an additional consequence or collateral effect of extensive carbapenem use may be the increased presence of SM, although more evidence and additional studies are warranted in this field. Thus, the design and implementation of carbapenem-focused antimicrobial stewardship programmes is a definite priority: on one hand, carbapenem-sparing (i.e., the use of non β-lactam drugs, especially in the context of ESBLs) and carbapenem-restriction (especially for empiric therapy, via pre-approval strategies and formulary restriction, according to specific criteria) interventions may be introduced [60, 61]; on the other other hand, if carbapenem use is unavoidable in the clinical situation, culture (to allow for de-escalation), precision antibiotic prescribing, adjustments for dose and duration with continuous laboratory support, and drug utilization studies are needed ensure the most rational use of these drugs [62, 63]. In addition, any and all interventions should be complemented by strict infection control measures, to ensure the prevention of HAIs.

Our study possesses some limitations that should be acknowledged: in addition to the retrospective, single-center study design, selection bias may affect our results, as our data originates from a tertiary-care center, where patients with more severe conditions or underlying illnesses are usually found. Our analyses used aggregated data only, individual patient data and turnover rates at specific wards were not collected, therefore the change in the number of patients in the relevant risk-populations for SM (e.g., cancer, immunosuppression, invasive surgery, ICU) was unknown. SXT resistance was determined based on routine methods of susceptibility testing, while the molecular survey of the resistance determinants was not performed in the isolates. During the analysis of systemic antibiotic use, some antibiotic groups (e.g., macrolides, imidazoles) were not analyzed individually, but grouped together as the „overall consumption of all other antimicrobial groups”, which was based on methodological considerations from previous studies [34, 35]. Finally, data from 2020 onwards was not included in our analyses, to remedy the potential bias to be introduced following the onset of the COVID-19 pandemic, and the extensive changes in antibiotic consumption and patient turnover rates.

## Conclusions

The present study reports on the epidemiology of SM, and the possible relationship between systemic antibiotic use and SM incidence at a tertiary-care teaching hospital over a 10-year surveillance period. Our results highlighted that increasing consumption of systemic antibiotics was associated with increasing SM and SXT-resistant SM incidence, with carbapenems, SXT, glycopeptides showing the strongest correlations, for both cases. Thus, implementation of institutional carbapenem-sparing strategies has a pivotal role in preserving these life-saving drugs, and may have major roles in affecting the microbial spectrum of infections in clinical settings.

## Funding

R.B. and M.G. were supported by the János Bolyai Research Scholarship of the Hungarian Academy of Sciences.

## Author contributions

Conceptualization: M.G.; Methodology: M.G.; R.B., M.M.; Data curation: R.B., M.M., Z.P., E.H.; Statistical analysis: M.G.; R.B., M.M.; Visualization: M.M.; Supervision: E.H.; Funding acquisition: R.B., M.M., Z.P.; Writing-original draft: M.G.; R.B., M.M.; Writing-review and editing: M.G.; R.B., M.M., Z.P., E.H.; All authors have read and agreed to the published version of the manuscript.

## Conflict of interest

The authors declare no conflict of interest, monetary or otherwise. The authors alone are responsible for the content and writing of this article.

## Data availability statement

All data generated during the study are presented in this paper.

## Abbreviations

AHI: antibiotic heterogeneity index
*bla*: beta-lactamase
COVID-19: coronavirus disease 2019
CR: carbapenem-resistant
CVC: central venous catheter
DDD: defined daily dose
ECCMID: European Congress of Clinical Microbiology and Infectious Diseases
ESBL: extended-spectrum β-lactamases
EUCAST: European Committee on Antimicrobial Susceptibility Testing
HAI: healthcare-associated infection
HC: Health Centre
HIV: human immunodeficiency virus
ICU: intensive care unit
MALDI-TOF MS: matrix-assisted laser desorption/ionization time-of-flight mass spectrometry
MDR: multidrug resistant
mini BAL: mini bronchoalveolar lavage
NEAK: National Health Insurance Fund of Hungary
NNSzR: National Nosocomial Infections Surveillance Network in Hungary
NSS: non-species specific
RND: resistance-nodulation-division
SARI: Surveillance of Antimicrobial Use and Antimicrobial Resistance in German Intensive Care Units
SD: standard deviation
SM: *Stenotrophomonas maltophilia*
SXT: sulfamethoxazole/trimetoprim
UK: United Kingdom
WHO: World Health Organization
XDR: extensively drug resistant
